# Donor-Derived Disseminated Toxoplasmosis in a Liver Transplant Recipient With Documented Sulfa Allergy

**DOI:** 10.1155/2024/8839805

**Published:** 2024-10-21

**Authors:** Zoe Raglow, Emily Herriman, Gabriel Abrams, Marc Najjar, Christopher J. Sonnenday, Kevin S. Gregg

**Affiliations:** ^1^Department of Internal Medicine, Division of Infectious Diseases, University of Michigan, Ann Arbor, Michigan 48109, USA; ^2^Department of Surgery, Division of Transplantation, University of Michigan, Ann Arbor, Michigan 48109, USA; ^3^School of Medicine, University of Michigan, Ann Arbor, Michigan 48109, USA; ^4^Center for Healthcare Outcomes and Policy, University of Michigan, Ann Arbor, Michigan 48109, USA

## Abstract

Transplant recipients who are seronegative for *Toxoplasma* and receive an organ from a seropositive donor are at high risk for donor-derived toxoplasmosis in the absence of prophylaxis. While the risk in cardiac transplant recipients is well known, this infection is often underrecognized in noncardiac transplant recipients. Toxoplasmosis in transplant patients is associated with high mortality, and diagnosis is challenging as the presentation is nonspecific. Recommendations for prophylaxis in cardiac transplant recipients are well-defined, but the optimal prophylactic strategy in noncardiac transplant recipients, especially those with sulfa allergies, is unknown. We report a case of donor-derived disseminated toxoplasmosis in a liver transplant recipient who did not receive prophylaxis due to documented sulfa allergy. The patient subsequently underwent a challenge with trimethoprim/sulfamethoxazole and was successfully treated with this therapy. This case underscores the variable clinical presentation of donor-derived toxoplasmosis as well as the critical importance of accurate allergy evaluation pretransplant.

## 1. Introduction

The parasite *Toxoplasma gondii* typically does not cause symptomatic infection in immunocompetent hosts, but in transplant patients can lead to severe, disseminated disease with very high mortality [1]. The nonspecific presentation of disseminated toxoplasmosis can make diagnosis challenging, necessitating a high degree of clinical suspicion. Recipients of solid organ transplants who are seronegative for *Toxoplasma* and receive an organ from a seropositive donor are at risk of toxoplasmosis; though this risk is well-documented in cardiac transplant recipients, data is scarce in recipients of other solid organ transplants. For this reason, recommendations on prophylaxis in this population are vague but generally include consideration of trimethoprim/sulfamethoxazole (TMP/SMX) for an undefined period posttransplant; recommendations on management of sulfa allergic patients are even less clear [2]. Despite the documented prevalence of sulfa allergies, the vast majority of these patients do not actually have hypersensitivity reactions and can safely receive sulfa drugs [3]. We present a case of disseminated, donor-derived toxoplasmosis in a liver transplant recipient with a documented sulfa allergy, review the literature on this topic, and suggest strategies for managing these patients.

## 2. Case Report

A 34-year-old male with a history of alcohol-related liver disease underwent a deceased donor liver transplant in September 2023. Relevant serologic testing included cytomegalovirus (CMV) D+/R−, Epstein–Barr virus (EBV) D+/R+, *Toxoplasma* D+/R−, and hepatitis B virus (HBV) core D+/R−. He did not receive induction immunosuppression, and he was discharged on tacrolimus 4 mg twice daily, mycophenolate mofetil 1000 mg twice daily, and prednisone 20 mg daily, as well as valganciclovir 900 mg daily, fluconazole 100 mg daily, inhaled pentamidine ×1 dose (due to documented sulfa allergy), and entecavir 0.5 mg daily for prophylaxis. His posttransplant course was complicated by acute kidney injury requiring hemodialysis, with renal recovery prior to discharge. The length of stay for index transplant admission was 2 weeks.

On Day 30 posttransplant, the patient noted a low-grade fever of 37.2°C, worsening fatigue, headache, and diarrhea. A gastrointestinal PCR panel was negative, and he was empirically started on amoxicillin–clavulanate 875–125 mg PO BID ×5 days. His symptoms did not improve, and he was admitted to the hospital on Day 34 posttransplant. On presentation, his temperature was 38.3°C, blood pressure 124/80 mm Hg, pulse 125 bpm, respiration 20 bpm, and oxygen saturation 98% on ambient air. He appeared ill; the exam was otherwise notable for a distended abdomen with a positive fluid wave. He had no nuchal rigidity. Laboratory data was significant for a white blood cell count of 4.5 K/*μ*L, hemoglobin of 7.8 g/dL, platelet count of 106 K/*μ*L, and creatinine of 2.17 mg/dL. Liver function was normal. A computed tomography (CT) scan of the chest showed patchy areas of consolidation in the right middle and bilateral lower lobes, trace right pleural effusion, and small pericardial effusion. CT abdomen/pelvis showed moderate volume ascites and gas-filled dilated small bowel and colon without transition point. CT head was unremarkable. A transthoracic echocardiogram showed a moderate pericardial effusion without evidence of tamponade. The patient was started on empiric antibiotics with piperacillin–tazobactam and vancomycin.

*Toxoplasma* PCR from blood was sent on hospital day 2, and the patient was empirically started on clindamycin (due to sulfa allergy), pyrimethamine, and leucovorin for suspected donor-derived disseminated toxoplasmosis.

**Figure 1:**
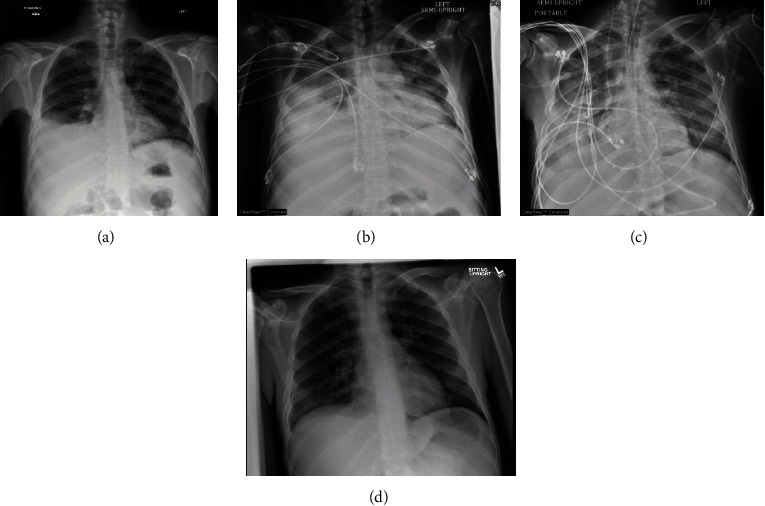
Serial chest X-rays (CXR) showing progression of parenchymal opacities consistent with ARDS. (a) Day of admission, with mild parenchymal opacities at the R lung base. (b) Hospital day 4, with worsening bilateral parenchymal opacities. (c) Hospital day 6, with ongoing bilateral parenchymal opacities. (d) Hospital day 45, parenchymal opacities have resolved.

On hospital day 3, the patient developed worsening hypoxemia and tachypnea and was intubated; he subsequently developed escalating oxygen requirements up to 100% FiO2, and inhaled nitric oxide was added. He was paralyzed and placed in a prone position. He was persistently febrile and tachycardic. Renal function worsened, and he was started on continuous renal replacement therapy. Serial chest X-rays showed worsening bilateral parenchymal opacities consistent with acute respiratory distress syndrome (ARDS, Figure 1). On day 4, *Toxoplasma* PCR returned positive at 18,700 copies/mL. *Toxoplasma* IgM was negative. Other workup, including blood, urine, and ascitic fluid cultures, plasma PCR for CMV, EBV, and adenovirus, and serum and urine antigens for *Cryptococcus*, *Histoplasma*, and *Blastomyces*, was negative. *Strongyloides* IgG was negative. Cell-free DNA sequencing (Karius test) was positive only for *Toxoplasma* (162,082 DNA molecules/*μ*L). MRI brain was performed on hospital day 15 (delayed due to patient instability) and was unremarkable.

Given clinical worsening despite therapy, allergy was consulted to evaluate the appropriateness of TMP/SMX therapy. The patient's documented reaction to TMP/SMX was hives. He underwent a 10/90 challenge with IV TMP/SMX, which he tolerated well, and was subsequently transitioned to TMP/SMX monotherapy at 5 mg/kg every 8 h equivalent (dosed for renal function) on hospital day 8. On hospital day 10, he started to improve with lessening oxygen requirements and was extubated to a nasal cannula on hospital day 12. Repeat *Toxoplasma* PCR decreased to 2900 copies/mL on hospital day 10 and was undetectable on hospital day 17. The patient was discharged to inpatient rehab on hospital day 28 and to home on day 44. He was treated with 6 weeks of induction therapy with high-dose TMP/SMX and subsequently transitioned to suppressive TMP/SMX. Six months posthospitalization, he has returned to baseline function with no evidence of recurrent infection.

## 3. Discussion

*Toxoplasma gondii*, the causative agent of toxoplasmosis, is a parasite acquired via consumption of undercooked meat containing tissue cysts or ingestion of oocytes shed in cat feces. Infection is typically asymptomatic in immunocompetent hosts, though rarely it can cause flu-like symptoms or organ involvement, and persists for life due to an encysted organism in host tissue. An estimated 11% of the US population is infected, though the prevalence is much higher in other countries [4]. *Toxoplasma* can also be transmitted via organ transplantation; the risk is highest in cardiac transplant recipients, but there are many reports of donor-derived toxoplasmosis in noncardiac solid organ transplant recipients as well [5].

The clinical manifestations of donor-derived toxoplasmosis are nonspecific and varied and may include fever, cough, headache, confusion, diarrhea, and focal neurologic symptoms. Patients often present with disseminated disease with multisystem organ involvement, including CNS involvement (meningitis or focal abscesses), pneumonitis, myocarditis, and chorioretinitis; mortality is estimated at > 50% [1]. Infection typically presents in the first 3–6 months posttransplant, but there are case reports of donor-derived toxoplasmosis presenting up to a year after transplant [6]. Because the presentation is often vague and easily confused with other common posttransplant infections, a high degree of clinical suspicion is needed for diagnosis. Given this diagnostic difficulty and high mortality, periodic surveillance using quantitative *Toxoplasma* PCR is recommended in high-risk (R+) hematopoietic stem cell transplant (HSCT) recipients, and preemptive therapy has been shown to improve outcomes in these patients [7]. Small studies in SOT patients have shown that PCR screening can detect early or asymptomatic infection, suggesting that this might be an effective strategy in high-risk SOT recipients as well, though given the paucity of data, this approach is not recommended by current guidelines [8, 9].

Since 2016, the Organ Procurement and Transplantation Network (OPTN) has mandated serologic screening of all organ donors for *Toxoplasma*, and current guidelines recommend screening all recipients as well [2, 10]. Because D+/R− cardiac transplant recipients are at high risk, the recommendation for lifelong TMP/SMX prophylaxis in these patients is longstanding. Despite universal screening and many reports of fatal cases of donor-derived toxoplasmosis in noncardiac transplant recipients, the optimal prophylaxis strategy in this population is unknown. Guidelines recommend consideration of TMP/SMX prophylaxis, but the evidence is poor, and duration is not well defined. Prophylaxis for patients with sulfa allergies is even less certain; the recommendation is to consider dapsone/pyrimethamine/leucovorin in these patients if glucose-6-phosphate dehydrogenase (G6PD) is negative, but this regimen is riddled with adverse effects and adds a significant pill burden [2]. In HSCT recipients, guidelines recommend atovaquone as another alternative in patients who cannot receive TMP/SMX [7]. There is some limited evidence of atovaquone's prophylactic efficacy in this patient population, but there are also many reports of breakthrough infections in high-risk patients [11–13]. In SOT recipients, there is virtually no data on prophylactic atovaquone use, and its efficacy is unknown.

Sulfa allergies are reported in about 3%–8% of the population, making this class one of the most common causes of drug-related allergic reactions. However, only a small fraction of these (an estimated 3%) are thought to be true hypersensitivity reactions [3]. TMP/SMX is the first-line agent for prophylaxis for not only *Toxoplasma* but also *Pneumocystis*; alternative prophylactic regimens are known to be inferior [14]. Protocols for delabeling and/or desensitizing of sulfa allergies in transplant recipients have demonstrated significant success, with most patients tolerating the drug without adverse reaction [15, 16].

In our case, the patient had a reported sulfa allergy (hives) but tolerated TMP/SMX. Evaluation by allergy pretransplant would likely have prevented the development of donor-derived toxoplasmosis as the patient would have received appropriate prophylaxis. Guidelines for the management of noncardiac transplant recipients with sulfa allergies who are at risk for donor-derived toxoplasmosis are vague due to a lack of available data, but the consequences of withholding prophylaxis can be dire. This case highlights the importance of considering toxoplasmosis in at-risk noncardiac organ transplant recipients and the varied and severe presentation of donor-derived toxoplasmosis, but also the critical importance of accurate and timely review of drug allergies in these patients.

## Data Availability

Data sharing is not applicable to this article as no new data were created or analyzed in this study.

## Nomenclature

ARDS: acute respiratory distress syndrome
CMV: cytomegalovirus
CT: computed tomography
EBV: Epstein–Barr virus
G6PD: glucose-6-phosphate dehydrogenase
HBV: hepatitis B virus
HSCT: hematopoietic stem cell transplant
OPTN: Organ Procurement and Transplantation Network
TMP/SMX: trimethoprim/sulfamethoxazole
